# High titers of antinuclear antibody and the presence of multiple autoantibodies are highly suggestive of systemic lupus erythematosus

**DOI:** 10.1038/s41598-022-05807-6

**Published:** 2022-02-01

**Authors:** Hejun Li, Yiqing Zheng, Ling Chen, Shunping Lin

**Affiliations:** Department of Rheumatology, Fujian Medical University Union Hospital, Fuzhou, China

**Keywords:** Diagnostic markers, Predictive markers, Systemic lupus erythematosus

## Abstract

The aim of this study is to evaluate the relationship between antinuclear antibody (ANA) titer and specificity, as well as the relationship between the number of positive-autoantibodies (AAbs) in antinuclear antibodies (ANAs) and specificity for systemic lupus erythematosus (SLE), so as to explore their significance in the diagnosis of SLE. A total of 1297 patients with ANA results was enrolled in this study, including 148 patients with SLE patients. The sensitivity, specificity, sensitive likelihood ratio and specific likelihood ratio of indicators in SLE were determined by receiver–operator characteristic (ROC) curve after measurement of ANA and ANAs by indirect immunofluorescence (IIF) and immunoblotting, respectively. ROC analysis showed that the specificity of ANA titer ≥ 1 +, ≥ 2 + and ≥ 3 + for SLE was estimated to be 81.29%, 90.69% and 96.52% respectively, with a increased titer-specific likelihood ratio (5.16, 9.29 and 19.60, respectively). The specificity of the number of positive-AAbs ≥ 1, ≥ 2 and ≥ 3 in ANAs for SLE was estimated to be 80.42%, 94.95% and 99.3% respectively, with a increased number-specific likelihood ratio (4.8, 15.26 and 72.48, respectively). The estimated sensitivity of the number of positive-AAbs ≥ 3, AnuA and anti-rRNP was higher than that of anti-Sm (p < 0.01) (50.68%, 41.89% and 31.76% vs. 16.89%, respectively), while there was no significant difference in their specificity (99.3%, 99.74% and 99.56% vs. 99.74%, respectively) (*p* > 0.05). High titers of ANA and the presence of multiple AAbs in ANAs are highly specific for SLE and highly suggestive of SLE. The likelihood of SLE can be assessed by ANA titer and the number of positive-AAbs in ANAs.

## Introduction

Systemic lupus erythematosus (SLE) is a prototypic autoimmune disease with highly variable clinical and immunological manifestations^1^, with a high rate of moderate and severe damage in young lupus patients^2^. Among these manifestations, the production of antibodies to components of the cell nucleus (antinuclear antibodies or ANAs) is a prominent serological finding. These autoantibodies (AAbs) target DNA, RNA, proteins and protein–nucleic acid complexes, with AAbs to DNA and Sm, a complex of proteins and uridine-rich RNA molecules, frequently emerged in SLE patients^3^. Because ANAs were originally discovered in patients with SLE, these AAbs have been considered a key if not invariable immunological finding. As such, the presence of ANAs has been considered a criterion in the classification of patients with SLE in either the American College of Rheumatology (ACR) or the Systemic Lupus International Collaborating Clinics (SLICC) criteria set^4,5^. A positive antinuclear antibody (ANA) is even required for further consideration for classification in 2019 European League Against Rheumatism (EULAR)/ACR Classification Criteria for SLE^6^. Because the high frequency of false positivity of ANAs has long been established, ANA is often considered as a screening indicator and is considered to lack specificity for SLE. But this is not entirely true. The low specificity of ANA is due to the low titer of cut-off we used. ANA is not only a dichotomous result (negative vs positive), but also different titers of ANA can provide additional help for the diagnosis of SLE. This paper elaborates the relationship between ANA titer and specificity, as well as the relationship between the number of positive-AAbs in ANAs and specificity for SLE through analysis of the sensitivity, specificity, sensitive likelihood ratio (negative likelihood ratio) and specific likelihood ratio (positive likelihood ratio).

## Materials and methods

### Patients

We retrospectively reviewed the medical records of patients whose ANA spectrum were examined in Fujian Medical University Union Hospital from August 2012 to August 2013. All enrolled SLE patients met SLICC Classification Criteria for SLE and misdiagnosed cases were ruled out by comprehensive clinical analysis. The remaining patients with ANA spectrum served as the control group. The exclusion criteria were defined as follows: (1) cases with uncertain diagnosis; (2) missing important data; (3) overlap syndrome. Ethical approval was obtained from the ethics board of Fujian Medical University Union Hospital. We confirm that all methods were performed in accordance with the relevant guidelines and regulations.

### Methods

ANA was detected by the indirect immunofluorescence assay on HEp2 cells (HEp2-IFA), and ANAs, including 13 antibodies (anti-U1 ribonucleoproteins (anti-U1RNP), anti-Sm antibody, anti-nucleosome antibody (AnuA), anti-ribosome ribonucleoprotein antibody (anti-rRNP), anti-dsDNA antibody, anti-histone antibody (AHA), anti-SSA antibody, anti-SSB antibody, anti-Scl-70 antibody, anti-PM-Scl antibody, anti-Jo-1 antibody, anti-CENP B antibody, and anti-PCNA antibody), was detected by immunoblotting, using the Euroimmun kit (Euroimmun (Beijing) Medizinische Labordiagnostika AG, China). Results are presented as negative (−), weak positive (±), and positive (1 +, 2 +, 3 +, 4 +). According to the instructions, ANA titer ≥ 1 + is defined as ANA positivity (above the laboratory reference range and consistent with SLICC SLE classification criteria).

In our study, the sensitivity of ANA greater than or equal to a certain titer is defined as the ratio of the number of SLE patients with this titer of ANA (true positive) to the number of all SLE patients (true positive + false negative).

The sensitivity of positive-AAbs greater than or equal to an amount was defined as the ratio of the number of SLE patients with this amount of positive-AAbs (true positive) to the number of all SLE patients (true positive + false negative).

The specificity of ANA greater than or equal to a certain titer is defined as the ratio of the number of non-SLE patients with ANA lower than this titer (true negative) to the number of all non-SLE patients (true negative + false positive).

The specificity of positive-AAbs greater than or equal to an amount is defined as the ratio of the number of non-SLE patients with positive-AAbs lower than this amount (true negative) to the number of all non-SLE patients (true negative + false positive).

### Statistical analyses

MedCalc was used for descriptive analysis on positive distribution of each indicator in each group; The ROC curve (receiver–operator characteristic curve) was used to analyze sensitivity, specificity, specific likelihood ratio and sensitive likelihood ratio of indicators in the diagnosis of SLE; differences in age at diagnosis between groups were compared by one-way ANOVA. Differences in gender, ANA-positive rate, ANA titer and the number of positive-AAbs in ANAs between groups and differences in sensitivity and specificity between various indicators were compared by Chi-square test.

### Informed consent

Informed consent was waived due to retrospective nature of the study.

### Ethics approval and consent to participate

The study was approved by the Ethical Committee of the “Fujian Medical University Union Hospital” in compliance with the ethical principles.

## Results

### Study population and characteristics

Finally, a total of 1297 patients with ANA results were included in the study. Among them, we identified 148 SLE patients (25 men and 123 women, mean age 35.14 years) as the research group, and 317 patients with non-SLE rheumatic diseases (including other autoantibody-associated rheumatic diseases, primary vasculitis, spondyloarthritis, osteoarthritis and metabolic joint disease), 99 patients with nephropathy (including proteinuria, hematuria and renal insufficiency not associated with SLE), 210 patients with hematological diseases (including leukemia, MDS, lymphoma, chronic myeloproliferative disorders and non-autoimmune induced anemia, thrombocytopenia, or leucopenia), and 523 patients with other diseases (including oncological, infectious, nervous and cardiovascular diseases)were included in the control group. Compared with other groups, SLE patients have a higher proportion of women and younger age, as shown in Table 1.

**Table 1:** The demographic characteristics between groups.

	SLE group (n = 148)	Non-SLE rheumatic diseases group (n = 317)	Hematological diseases group (n = 210)	Nephropathy group (n = 99)	Other control group (n = 523)
Gender, F (%)	123* (83.11)	229 (72.24)	114 (54.29)	46 (46.46)	277 (52.96)
Age at diagnosis, year, mean ± SD	35.14 ± 14.27^▲^	44.28 ± 18.25	41.64 ± 20.54	41.78 ± 18.08	44.33 ± 20.91

*p < 0.05, compared with Non-SLE rheumatic diseases group, Nephropathy group, Hematological diseases group, and Other control group.

^▲^p < 0.01, compared with Non-SLE rheumatic diseases group, Nephropathy group, Hematological diseases group, and Other control group.

*SLE* systemic lupus erythematosus.

### Comparison of ANA titers between groups

ANA- positive rates (ANA titer ≥ 1 +) in SLE group and non-SLE rheumatic diseases group were 96.62% and 50.47%, respectively, and the proportions of ANA 2 + or above (ANA titer ≥ 2 +) in the two groups were 86.49% and 30.91%, respectively, which were significantly higher than those in nephropathy group (6.06%, 0), hematological diseases group (10%, 0), or other diseases group (5.35%, 1.72%) (*p* < 0.01). Moreover, the proportions of ANA 3 + or above (ANA titer ≥ 3 +)in SLE was 68.24%, which was significantly higher than that in non-SLE rheumatic diseases group (11.67%) (*p* < 0.01), as shown in Table 2.

**Table 2:** Comparison of the proportions of patients with different levels of ANA titers between groups.

ANA titers	SLE group (n = 148)	Non-SLE rheumatic diseases group (n = 317)	Nephropathy group (n = 99)	Hematological diseases group (n = 210)	Other diseases group (n = 523)
−, n (%)	0	113 (35.65)	76 (76.77)	129 (61.43)	393 (75.14)
±, n (%)	5 (3.38)	44 (13.88)	17 (17.17)	60 (28.57)	102 (19.50)
≥ 1 +, n (%)	143 (96.62*)	160 (50.47*)	6 (6.06)	21 (10.00)	28 (5.35)
≥ 2 +, n (%)	128 (86.49*)	98 (30.91*)	0	0	9 (1.72)
≥ 3 +, n (%)	101 (68.24^▲^)	37 (11.67)	0	0	3 (0.57)
≥ 4 +, n (%)	11 (7.43)	1 (0.32)	0	0	0

± : weak positive.

*p < 0.01, compared with Nephropathy group, Hematological diseases group, and Other diseases group ^▲^p < 0.01, compared with Non-SLE rheumatic diseases group.

*ANA* antinuclear antibody, *SLE* systemic lupus erythematosus.

### Comparison of the number of positive-AAbs in ANAs between groups

ANAs- positive rates (the number of positive-AAbs ≥ 1 in ANAs) in SLE group and non-SLE rheumatic diseases group were 93.92% and 42.59%, respectively, and the proportions of two or more positive-AAbs (the number of positive-AAbs ≥ 2) in the two groups were 77.03% and 14.83% respectively, which were significantly higher than those in nephropathy group (5.05%, 0), hematological diseases group (14.29%, 2.38%), or other diseases group (10.52%, 1.15%) (p < 0.01). The proportions of three or more positive- AAbs (the number of positive-AAbs ≥ 3) in SLE group was 50.68%, which was significantly higher than that in non-SLE rheumatic diseases group (2.52%) (p < 0.01), as shown in Table 3.

**Table 3:** Comparison of the proportions of patients with different numbers of AAbs between groups.

The number of positive-AAbs in ANAs	SLE group (n = 148)	Non-SLE rheumatic diseases group (n = 317)	Nephropathy group (n = 99)	Hematological diseases group (n = 210)	Other diseases group (n = 523)
0, n (%)	9 (6.08)	182 (57.41)	94 (94.95)	180 (85.71)	467 (89.29)
0.5, n (%)	0	0	0	0	1 (0.19)
≥ 1, n (%)	139 (93.92*)	135 (42.59*)	5 (5.05)	30 (14.29)	55 (10.52)
≥ 2, n (%)	114 (77.03*)	47 (14.83*)	0	5 (2.38)	6 (1.15)
≥ 3, n (%)	75 (50.68^▲^)	8 (2.52)	0	0	0
≥ 4, n (%)	48 (32.43)	4 (1.26)	0	0	0

0.5: weak positive.

*p < 0.01, compared with Nephropathy group, Hematological diseases group, and Other diseases group ^▲^p < 0.01, compared with Non-SLE rheumatic diseases group.

*AAbs* auto-antibodies, *ANAs* antinuclear antibodies, *SLE* systemic lupus erythematosus.

### The specificity of ANA titer, the number of positive-AAbs in ANAs and various AAbs for SLE

**Figure 1:**
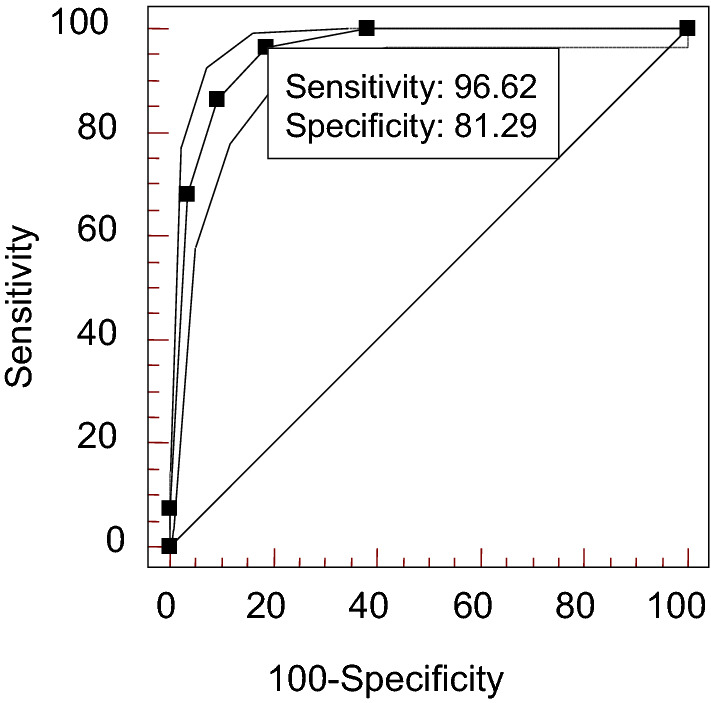
ROC curve analysis for ANA titer. *ANA* antinuclear antibody.

**Figure 2:**
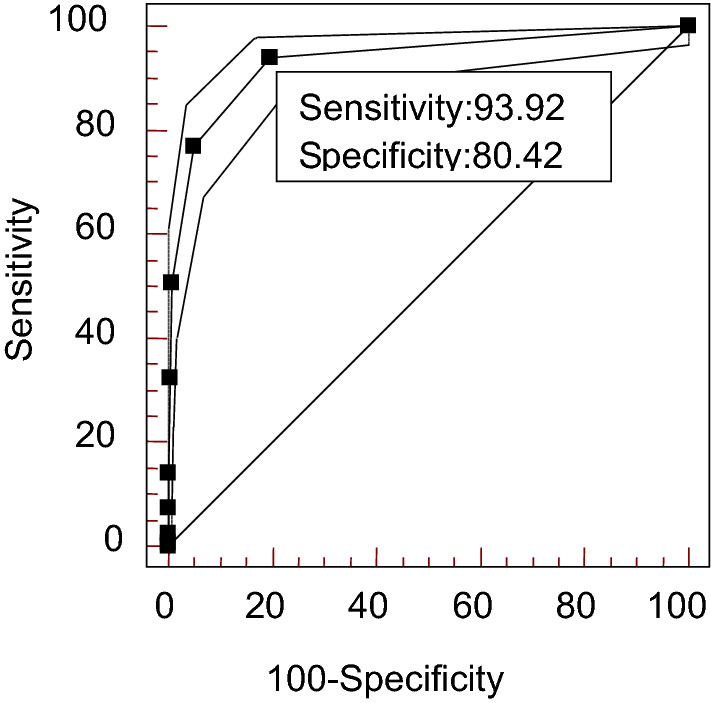
ROC curve analysis for the number of positive-AAbs. *AAbs* autoantibodies.

The sensitivity, specificity, likelihood ratio and the area under the ROC curve of each index are shown in Table 4. The area under the ROC curve of ANA titer and the number of positive-AAbs was 0.954 and 0.933, as shown in Figs. 1 and 2. The specificity of ANA titer ≥ 1 + for SLE was estimated to be 81.29%, with a high sensitivity (96.62%), low estimated specific likelihood ratio (5.16), and low estimated sensitive likelihood ratio (0.042). The estimated specificity increased to 90.69% and 96.52%, and the estimated titer-specific likelihood ratio increased to 9.29 and 19.60 for a titer of ≥ 2 + and ≥ 3 + respectively.

**Table 4:** The specificity of ANA titer, the number of positive-AAbs in ANAs and various AAbs for SLE.

	Sensitivity (%)	Specificity (%)	+ LR	− LR	The area under the ROC curve (Az)
**ANA titer**					
≥ 1 +	96.62	81.29	5.16	0.042	0.954
≥ 2 +	86.49	90.69	9.29	0.15	
≥ 3 +	68.24	96.52	19.6	0.33	
**The number of positive-AAbs in ANAs**					
≥ 1	93.92	80.42	4.8	0.076	0.933
≥ 2	77.03	94.95	15.26	0.24	
≥ 3	50.68*	99.3^▲^	72.78	0.5	
Anti-U1 RNP	41.22	98.0	20.59	0.6	0.705
Anti-Sm	16.89	99.74	64.7	0.83	0.599
AnuA	41.89*	99.74^▲^	160.45	0.58	0.715
Anti-rRNP	31.76*	99.56^▲^	72.98	0.69	0.662
AHA	41.89	98	20.95	0.59	0.702
Anti‐dsDNA	13.51	98.87	11.94	0.87	0.567
Anti-SSA	63.51	87.47	5.07	0.42	0.767
Anti-SSB	22.97	97.21	8.25	0.79	0.606

*p < 0.01, compared with anti-Sm. ^▲^*p* > 0.05, compared with anti-Sm.

*ANA* antinuclear antibody, *ANAs* antinuclear antibodies, *AAbs* auto-antibodies, *SLE* systemic lupus erythematosus, + *LR* the estimated positive likelihood ratio, −*LR* the estimated negative likelihood ratio, *anti-U1RNP* anti-U1 ribonucleoproteins, *anti-Sm* anti-Sm antibody, *AnuA* anti-nucleosome antibody, *anti-rRNP* anti-ribosome ribonucleoprotein antibody, *anti-dsDNA* anti-dsDNA antibody, *AHA* anti-histone antibody, *anti-SSA* anti-SSA antibody, *anti-SSB* anti-SSB antibody.

The specificity of the number of positive-AAbs ≥ 1 in ANAs for SLE was estimated to be 80.42%, with a high sensitivity (93.92%), low estimated specific likelihood ratio (4.8) and low estimated sensitive likelihood ratio (0.076). The estimated specificity increased to 94.95% and 99.3%, and the estimated number-specific likelihood ratio increased to 15.26 and 72.48 for the number of positive-AAbs ≥ 2 and ≥ 3.

The estimated sensitivity of the number of positive-AAbs ≥ 3, AnuA and anti-rRNP was higher than that of anti-Sm (*p* < 0.01) (50.68%, 41.89% and 31.76% vs. 16.89%, respectively), while there was no significant difference in their specificity (99.3%, 99.74% and 99.56% vs. 99.74%, respectively) (*p* > 0.05) (Table 4).

## Discussion

As is known to all, SLE patients are mostly young women of reproductive age, and our study is no exception. Therefore, SLE is an important field worth of study. The basic pathogenesis of systemic lupus erythematosus (SLE) is the immune imbalance that causes the generation of a variety of pathogenic AAbs in vivo. The latter often has existed for several years before the first clinical symptom of the disease. AAbs are closely related to the target tissue damage in SLE patients^7,8^, so they are correlated with clinical manifestations of SLE patients and significant for the diagnosis of SLE and determination of disease activity.

However, as the two main features of SLE, multi-system damage and AAbs are not unique to SLE because they can also be present in patients with infections, tumors and chronic diseases, especially in the elderly. So, although there are many criteria for SLE diagnosis, all of them are classification criteria, rather than diagnostic criteria. That is, patients who even meet the criteria such as the SLICC classification criteria for a diagnosis of SLE are not necessarily SLE. Therefore, while taking advantage of high sensitivity of low titers of ANA to include suspected patients into diagnostic consideration, understanding specificity of high titers of ANA can help improve the accuracy of SLE diagnosis, reduce misdiagnosis and make clinicians more certain in SLE diagnosis, especially in complicated patients.

In this study, clinical data of SLE patients initially diagnosed within a year in our hospital and other patients in control group were collected to analyze the relationship between ANA titer and specificity, as well as the relationship between the number of positive-AAbs in ANAs and specificity through analysis of the sensitivity, specificity, sensitive likelihood ratio and specific likelihood ratio.

Although there was different experimental method to detect ANA^9^, IFA is still considered the gold standard for ANA screening^10^. At a low cutoff of ANA titer 1 +, ANA has a high sensitivity (96.62%) but low specificity (81.29%), similar to previous reports^9,11^. The high sensitivity of low cutoff helps to distinguish patients as ANA positive or negative and screens suspected ANA-associated systemic rheumatic disease (AASRD) patients from a large number of patients. However, that's not enough, because a significant proportion of patients with ANA titer 1 + also appear in the control group, as shown in Table 2. Given a low prevalence of AASRD, these false-positive results may trigger unnecessary additional analyses. Given the importance of ANAs in the diagnosis of AASRD, in-depth understanding of ANAs is conducive to reduce a delay in treatment, a wrong diagnosis—either through false positive or false negative tests, which may also be responsible for additional costs due to the repetition of confirmatory tests and/or to consequent unnecessary diagnostic investigations. As mentioned above, ANA tests not only provide negative or positive results, but also provide different titers. This additional information may be important for us to identify related diseases. Our study showed that most SLE patients (86.49%) had an ANA titer of ≥ 2 +, in sharp contrast to non-rheumatic controls. Higher titers of ANA (ANA titer ≥ 3 +) were also rare in non- SLE rheumatic patients (11.67%) (Table 2). ROC analysis showed that as the ANA titer increased, the specificity and specific likelihood ratio increased correspondingly. A high titer of ANA (≥ 3 +) was Highly specific for SLE (96.52%) with a high specific likelihood ratio (19.6), enough to predict the diagnosis of SLE. These data clearly indicate that when a high titer of ANA is present, the diagnostic accuracy of clinicians can be improved, and there is no need for too many tests to distinguish SLE from other diseases, which can reduce unnecessary economic costs, since the possibility of a false positive with a high titer of ANA is minimal. In addition, ANA does not have a 100% positive rate in SLE, so when there is a classical SLE clinical manifestation, we can not rule out the possibility of SLE when ANA is negative.

AAbs have an extremely important role in the pathophysiology of SLE. A variety of antibodies against autoantigens can be produced in SLE patients, resulting in damage to multiple organs in patients with SLE. In our study, thirteen AAbs could be detected by Immunoblotting. The high positive rate of multiple AAbs (77.03% of AAbs ≥ 2 and 50.68% of AAbs ≥ 3) in ANAs of SLE group reflects the basic pathogenesis of the highly disordered immunity in SLE patients that generates a variety of AAbs. The sensitivity and specificity of single antibody for SLE have been studied frequently, while little attention has been paid to the number of positive-AAbs in the ANAs. Given the high positive rate of multiple AAbs in SLE patients, the number of AAbs may also provide additional information on the sensitivity and specificity of SLE diagnosis. We uniquely found that the number of positive-AAbs in ANAs had a similar relationship with the specificity for SLE as ANA. At a cutoff of the number of positive-AAbs ≥ 1 and ≥ 2, the specificity for SLE was estimated to be 80.42%, and 94.95%, and the specific likelihood ratio was estimated to be 4.8 and 15.26 respectively. The specific likelihood ratio of the number of positive-AAbs ≥ 3 was 72.78 in SLE diagnosis, which was highly suggestive of SLE. Meanwhile, the sensitive likelihood ratio for positive-AAbs was 0.076 in SLE diagnosis, which indicates low possibility of SLE when all AAbs are negative.

Anti-SM is well known as a retrospective marker antibody of SLE. Contrary with high sensitivity and low specificity of low titer of ANA for SLE diagnosis, anti-SM showed a low sensitivity (16.89%) and high specificity (99.74%), with a specific likelihood ratio of 64.75, suggesting that anti-Sm detected by immunoblotting had good positive predictive value for the diagnosis of SLE. However, due to low sensitivity, the diagnostic value for most negative patients is limited.

Nucleosome, a major autoantigen in SLE pathogenesis, can stimulate the body to produce antibodies. Previous studies showed that the specificity of AnuA detected by ELISA in SLE diagnosis is equal to or higher than that of anti-dsDNA, but its sensitivity differs in studies^12–14^. Anti-rRNP is also studied deeply in SLE and correlated with SLE activity^15^. In this study, the specificities of the number of positive-AAbs at a cutoff of ≥ 3, AnuA and anti-rRNP are similar to that of anti-Sm for SLE (*p* > 0.05), but their sensitivity are higher than that of anti-Sm (50.68%, 41.89% and 32.43% vs. 16.89%) (p < 0.01), suggesting that these three indicators, especially the number of positive-AAbs at a cutoff of ≥ 3, are better immunological indicators for SLE diagnosis than anti-Sm.

ANA spectrum is a commonly used and extremely important tool in rheumatism, and exploring new clinical significance is helpful to better grasp the diagnosis of SLE, and eventually avoid missed diagnosis, misdiagnosis and excessive medical costs.

## Conclusions

This study shows that the vast majority of SLE patients have positive ANA and AAbs in ANAs, and most patients have an ANA titer of ≥ 2 + and an amount of positive AAbs of ≥ 2. High titers of ANA and the presence of multiple AAbs in ANAs are highly specific for SLE, while low titers of ANA and positive-AAbs in ANAs are highly sensitive for SLE. Low titers of ANA and positive-AAbs are an effective screening index for SLE, while high titers of ANA and the presence of multiple AAbs are highly suggestive of SLE. The likelihood of SLE can be assessed by ANA titer and the number of positive-AAbs in ANAs.

## Data Availability

Available under request.
